# Molecular Crosstalk between Chromatin Remodeling and Tumor Microenvironment in Multiple Myeloma

**DOI:** 10.3390/curroncol29120749

**Published:** 2022-12-05

**Authors:** Chandraditya Chakraborty, Srimoyee Mukherjee

**Affiliations:** 1 Jerome Lipper Multiple Myeloma Center, Dana-Farber Cancer Institute, Harvard Medical School, Boston, MA 02215, USA; 2 Department of Developmental, Molecular and Chemical Biology, Tufts University School of Medicine, Boston, MA 02111, USA

**Keywords:** multiple myeloma, tumor microenvironment, chromatin remodeling

## Abstract

Multiple myeloma (MM) is a complex disease driven by numerous genetic and epigenetic alterations that are acquired over time. Despite recent progress in the understanding of MM pathobiology and the availability of innovative drugs, which have pronounced clinical outcome, this malignancy eventually progresses to a drug-resistant lethal stage and, thus, novel therapeutic drugs/models always play an important role in effective management of MM. Modulation of tumor microenvironment is one of the hallmarks of cancer biology, including MM, which affects the myeloma genomic architecture and disease progression subtly through chromatin modifications. The bone marrow niche has a prime role in progression, survival, and drug resistance of multiple myeloma cells. Therefore, it is important to develop means for targeting the ecosystem between multiple myeloma bone marrow microenvironment and chromatin remodeling. Extensive gene expression profile analysis has indeed provided the framework for new risk stratification of MM patients and identifying novel molecular targets and therapeutics. However, key tumor microenvironment factors/immune cells and their interactions with chromatin remodeling complex proteins that drive MM cell growth and progression remain grossly undefined.

## 1. Introduction

Multiple myeloma (MM) is a complex hematological malignancy that is characterized by clonal expansion of plasma cells within the bone marrow [1]. While the healthy plasma cells fight infections by producing antibodies, in multiple myeloma, cancerous plasma B cells accumulate in the bone marrow instead of the normal plasma cells and substitute the normal functioning of antibody production. MM is currently the second most common in the United States and constitutes about 13% of all hematological cancers [2]. In almost all MM patients, disease progression occurs by a specific sequence of events involving molecular modifications of plasma cells to its malignant form. This is followed by additional secondary mutation events, augmenting the disease progression. MM is a culmination of complex interplay between genetic aberration and cytogenetic alterations with the aberrations in the bone marrow environment, which results in modified immune system response and associated pathways [1], which essentially have an impactful role in chromatin remodeling. Chromatin remodeling is controlled by several chromatin remodeling complexes and some of these complex members, such as BCL7A and ARID family [3], have mutations in the noncoding and coding region of the myeloma genome. Recent studies have shown that ARID2, a component of the polybromo-associated BAF (PBAF) SWI/SNF complex, is a pomalidomide-induced neo substrate of CRL4^CRBN^ [4]. In addition to BRD7, another crucial subunit of PBAF SWI/SNF complex is critical for pomalidomide-induced ARID2 degradation, indicating the therapeutic intervention of MM through SWI/SNF chromatin remodeling factors [4]. Additionally, the tumor microenvironment (TME) plays an immensely important role in promoting the expansion of specialized plasma cell types with the most aggressive traits. The bone marrow (BM) microenvironment contains various components, including the tumor-associated macrophages (TAMs), and has recently gained interest as a potential therapeutic target. In this review, we will provide updated and comprehensive insight into the current knowledge on the role of TME and its component immune cells, as well as its effect and crosstalk with chromatin remodeling factors and complexes.

## 2. Chromatin Remodeling and Myeloma

Chromatin is the basic and central structure in a cell which enables dynamic central regulation of cellular transcription. A large array of genomic components that are generally silent in the normal physiologic state are activated by an aberrant expression and/or hyperactivity of chromatin remodelers in cancer cells in response to dysregulated cellular signals [5]. Chromatin remodeling is grossly a rearrangement of the basic chromatin structure by sequential repositioning of the nucleosome. The basic nucleosome mainly is made up of histone core proteins (H2A, H2B, H3, and H4) and followed by wrapping up by a 150-bp DNA sequence, which is affected in number of ways, including molecular events, such as nucleosome ejection, nucleosome sliding, and histone eviction [5]. Nucleosome sliding represents the movement of the histone octamer across the DNA sequence, whereas, on the other hand, nucleosome ejection refers to the total segregation of the histone core protein from the interacting DNA molecule. On the other hand, histone eviction primarily deals with the removal or replacement of H2A–H2B dimer proteins as a molecular effect of disintegration of the core histone octamer protein [5]. Basically, nucleosome ejection, sliding, and removal of the H2A–H2B dimers directly affects DNA and nucleosome stability adversely, thus playing a major role in transcriptional machinery and gene regulation of the cell [6]. In chromatin remodeling, SWI/SNF complexes are essential for cellular differentiation and proliferation but, unfortunately, the molecular mechanisms of the functionality of the several chromatin remodelers are not well studied and need in-depth experimental approaches to uncover their role in myeloma. Several biochemical analyses of SWI/SNF complex show it contains several DNA-binding domains, nucleosome-binding domains, high-mobility group box domains, and, finally, histone protein-binding domains (e.g., bromodomains and plant homeodomains) [7]. 

Recent high-throughput molecular and crystallography studies have identified three distinct mammalian SWI/SNF (m-SWI/SNF) complexes: canonical-BAF, p-BAF (polybromo-associated BAF), and noncanonical BAF (ncBAF). All of these three complexes contain a mutually exclusive subunit, SMARCA4 or SMARCA2. These structures showed the first mechanistic insights into ATP-driven rearrangements causing histone eviction [7]. Since the basic fundamental role of m-SWI/SNF complexes is maintenance and accessibility of prime transcription factors, as a molecular consequence, they also exert a considerable antitumor effect. Consequently, m-SWI/SNF-perturbated cellular state can trigger reprograming of cellular processes and can also drive oncogenic pathways. Loss-of-function mutations in genes encoding SWI/SNF subunits are found in about >20% of human cancers, with point mutations occurring about twice as often as deletions [7]. Much research led to the discovery of chromatin remodelers and complexes, such as ATP-dependent macromolecular machineries, or remodelers, such as the nucleosome remodeling and histone deacetylase complex (NuRD), nucleosome remodeling factor (NURF), chromatin assembly complex (CHRAC), and the most important SWI/SNF complex [8,9].

Multiple myeloma is characterized by genetic changes in the chromatin remodelers and regulators. Recent studies have shown mutation in chromatin regulators, such as KMT2C (14.3%), KMT2D (14.3%), EP300 (11.6%), and ARID gene family (31.3%), were observed to be frequently mutated in newly diagnosed MM (NDMM) patients [10]. Chromatin remodeling and histone rearrangement is characterized by popular chromatin remodelers, such as ARID family proteins (ARID1A, ARID1B, and ARID2) [11], and several important proteins, such as DNA methyl transferases, DNMT1 and DNMT3A; histone-modifying enzymes, such as HAT1, KMT2C, etc. [12], have a deep impact in the organization of myeloma genome, and aberration in any of these factors leads to change in clonal propagation of myeloma cells. However, since the mutation frequency of these factors is less frequent in myeloma, thorough investigation on the other genetic alterations and interactions with the immune effector cells in the microenvironment can be crucial in understanding the chromatin remodeling and microenvironment milieu in myeloma. Apart from chromatin regulators, genes encoding the chromatin remodeling SWI/SNF complex subunits are frequently mutated in about 16% in multiple myeloma, indicating the importance of this complex in MM progression [13]. Although several high-throughput molecular studies have identified epigenetic changes in the genome, their pathogenic impact is still unclear and the chromatin regulatory pathway directing abnormal cellular functions in MM is still under several investigations [13]. Most importantly, a very important study has shown that chromatin remodeling is affected by pathogenic factors of myeloma microenvironment leading to activation of chromatin and upregulation in genes involved in p53, NF-kB, and mTOR molecular pathways [14]. Thus, aberrant chromatin remodeling leading to its activation is a result of MM microenvironment.

## 3. Components of Myeloma Tumor Microenvironment (TME) and their Impact

**Figure 1:**
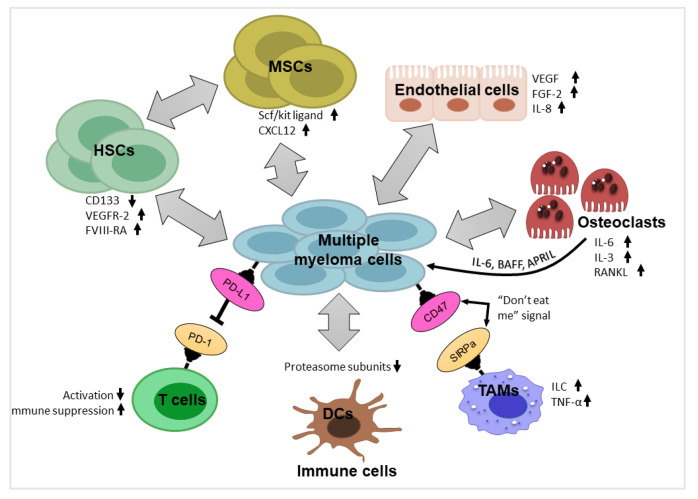
Impact of some critical components of tumor microenvironment on MM progression.

The myeloma bone marrow (BM) niche significantly acts as a pathogenic entity in MM, and the BM milieu has been reported to augment plasma cell growth and tumor cell progression and homing [15]. The BM milieu of MM patients varies in its genetic, epigenetic, and noncellular organization from that of normal plasma cells from normal subjects [16]. Tumor microenvironment, which controls MM progression, actually is part of the bone marrow, which is made up of the following essential cellular elements: extracellular matrix proteins (EMPs), hematopoietic stem cells (HSCs), progenitor cells, mesenchymal stromal cells (MSCs), endothelial cells, osteoblasts, growth factors, immune cells, etc. The TME of myeloma accommodates different types of immune cells, which persists in an immune-deprived condition and results in an abrogated immune response through deregulated immune cells and pathways (summarized in Figure 1). 

### 3.1. Hematopoietic Stem Cells and Progenitor Cells

Hematopoietic stem cells (HSCs) reside in the bone marrow and they give rise to all the blood cell types of the myeloid and lymphoid lineages [17]. In adults, HSCs and hematopoietic stem and progenitor cells (HSPCs) reside in the bone marrow, acting according to their maturation states and activity [18,19]. HSCs are believed to exist close to the arterioles and any stress generally leads to HSC proliferation and distribution at distant locations in the body [20,21,22]. HSCs and HSPCs of MM patients differentiate into cells with endothelial cell characteristics, whereby they tend to express less CD133 and develop VEGFR-2, vascular endothelial (VE)-cadherin and factor VIII-related antigen (FVIII-RA) [23]. HSCs and progenitors have an immense effect on determining how the niche supports the growth of tumor. An important study mentions that genomic profiling of HSCs and progenitor cell subsets revealed aberrations of MM microenvironment signaling pathways, such as TGFβ signaling, cellular migration, cellular adhesion, etc. [24]. Furthermore, inhibition of the involving factors of HSCs and progenitor cells depends on MM-associated microenvironment conditions [24]. 

### 3.2. Mesenchymal Stromal Cells

Mesenchymal stromal cells (MSCs) are a heterogenous population of cells having self-renewal properties and defined by markers, such as nestin, leptin receptor, neural-glial antigen (NG)-2, and paired related homeobox (Prx-1) [22,25,26,27]. MSCs can differentiate into different lineages, including osteoblastic cells, chondrocytes, and adipocytes [28]. MSCs associate with HSCs by secreting supporting elements, such as stem cell factor (SCF/Kit ligand), C-X-C motif chemokine ligand 12 (CXCL12), and others, which differs according to their location in the bone marrow microenvironment (BMM) [29].

### 3.3. Endothelial Cells

Endothelial cells line the vascular system and play crucial roles in regulating tumor initiation, progression, and metastasis. Angiogenesis is an important feature of tumor metastasis and tumor cells are receptive to growth factors specific for endothelial cells and result in a switch in the balance of positive and negative angiogenic factors [30]. The hypoxic environment of the bone marrow promotes acquisition of the epithelial–mesenchymal transition (EMT) machinery in MM cells, leading to their enhanced mobilization away from the BMM [31]. MM endothelial cells (MMEC) secrete proangiogenic factors, such as VEGF, FGF-2, and IL-8, by enhanced transcription by platelet-derived growth factor (PDGF)-BB/PDGF receptor beta (PDGFRβ), thus promoting the tumorigenesis [32]. In contrast, a prolonged treatment with a PDGFRβ/SrcTK inhibitor reduced the expression of endogenous VEGF, thus abrogating this effect. Additionally, endothelial cells have been shown to support HSC maintenance by providing factors, such as CXCL12, SCF, angiopoietin, fibroblast growth factor (FGF) 2, and Delta-like 1 [19,33]. Furthermore, removal of E-selectin from endothelial cells increased HSC quiescence and self-renewal, confirming that E-selectin also supports HSC function [34].

### 3.4. Immune Cells

Tumor-infiltrating lymphocytes (TILs) and tumor-associated macrophages (TAMs) are among the critical immune cells that have a pivotal role in the multiple myeloma TME and, thus, have long been considered as promising targets for therapeutic intervention.

The adaptive immune system is equipped with pivotal properties, such as recognition and elimination especially by CD8+ cytotoxic T lymphocytes (CTLs) [35]. It is known that upregulation of programed cell death-ligand 1 (PD-L1) on cancer cells inhibits immune intervention by binding to its specific receptor programed cell death protein 1 (PD1), expressed on activated T cells [36]. High PD-L1 expression is an indicator of malignant plasma cells, which makes it a promising target for immune checkpoint inhibitors (ICI) [37,38]. Additionally, MM is characterized by reduced T cell activation, resulting in an immunosuppressive environment, as shown by studies on BM of MM patients containing an increased number of PD1-positive and T cell immunoglobulin and mucin-domain-containing protein 3 (TIM3)-positive T cells [39]. Nevertheless, although ICI monotherapies have not shown clinical benefits to MM patients [40], combination therapy shows better efficacy and safety with advanced malignancy [41]. Dendritic cells (DCs) in the TME could, furthermore, protect the myeloma cells from CTL-mediated cell killing by downregulating expression of proteasome subunits [42].

Tumor-associated macrophages (TAMs) are known to promote disease progression in numerous solid tumors, including melanoma, glioblastoma, lung cancer, colorectal cancer, and ovarian cancer [43,44,45,46,47]. Many preclinical models have been utilized to study how TAMs affect MM cell survival. The proinflammatory cytokines (IL-6 and TNFα) secreted by CD169-positive TAMs are known to enhance vascular leakage and abrogate CD138-mediated cell adhesion; this may drive dissemination of myeloma cells into the blood circulation [48]. In tandem, M2-polarized macrophages are established to promote angiogenesis [49]. In a study, while xenografts in untreated mice displayed increased tumor growth and VEGF, they were reduced in myeloma xenografts injected with M2-polarized macrophages and treated with the macrophage-depleting agent clodronate [50]. TAMs have immense effect on angiogenesis, as a report suggests that the transcriptomics profile of the immune cells of MM patients represented enrichment of particular gene types, including VEGF-A or diphtheria toxin receptors [51]. TAMs exercise influence on myeloma cells by preventing elimination by the immune system and boosting their cell survival. It has been reported that M2 macrophages can prevent MM cells from apoptosis induced by drugs, possibly by enhanced resistance and tumor progression [52,53]. Reduced CXCR4 receptor was detected in MM patients undergoing bortezomib treatment, which can be an indication of impaired adhesion and increased expression of macrophage migration inhibitory factor (MIF) [54,55]. Increased expression of CD47 is another characteristic of MM patients and its binding to signal regulatory protein a (SIRPa) on macrophages generally act as a “don’t eat me signal” and safeguards myeloma cells from phagocytosis and apoptosis [56].

### 3.5. Osteoblasts and Osteoclasts

The effect of osteoblasts and osteoclasts on MM progression are less studied in comparison to the other components of the TME. In MM patients, osteoclast activation and osteoblast differentiation inhibition result in a mutual imbalance, which, in turn, impairs osteoblastogenesis [57,58,59]. This is the reason why MM patients often experience bone pain and fractures [60,61].

Osteoclasts influence MM progression directly by releasing cytokines, such as IL-6, IL-3, and others, or expressing receptor activator of NF-kB ligand (RANKL), which leads to resorption of the bone matrix [62,63,64,65]. This turns on the “vicious cycle” of MM growth by several soluble factors, such as IL-6, BAFF, APRIL, and others [66,67]. IL-6 downregulates CD138 and enhances the permeability of blood vessels, enabling intravasation of cancer cells and exerting an overall pro-tumorigenic role [48]. In contrast, osteoblasts balance the osteoclast activity by pushing MM cells towards the quiescent state and initiating apoptosis [68]. MM cells try to combat this effect by releasing secreted frizzled-related protein 2 (sFRP2), Dickkopf-related protein 1 (DKK1), and trans-forming growth factor beta (TGF-β) to conquer the tumor-suppressive outcomes of osteoblasts [64,69,70].

### 3.6. Adipocytes

Recently, bone metastatic cancers, such as breast cancer and acute myelogenous leukemia, have been linked to bone marrow adipocytes [71,72,73]. Some key risk factors in multiple myeloma disease prevalence are obesity and aging of the bone marrow, indicating that bone marrow adipose tissue (BMAT) may affect the progression of MM [74,75,76]. Thus, the mutual interactions between BMATs and myeloma cells have substantial effects on the pathogenesis and treatment of multiple myeloma and could be used as a potential therapeutic target for future interventions [77].

### 3.7. Extracellular Matrix Proteins

The extracellular matrix (ECM) is a critical component of the tumor microenvironment that contributes to the regulation of cell survival, proliferation, differentiation, and metastasis. In addition to myeloid cells that penetrate myeloma, extracellular matrix components and stromal cells also play a role [78]. One of the reports suggest that tumor ECM is reconstructed at the mRNA and protein levels in MGUS and MM to promote their progression; moreover, decreased survival in MM has been affiliated to two ECM proteins, ANXA2 and LGALS1 [79]. An extracellular matrix metalloproteinase inducer (EMMPRIN), CD 147, is also connected with MM development [80]. Therefore, the connection between MM cells and the bone marrow (BM) microenvironment, such as the ECM, is necessary to the pathogenesis of the sickness and the improvement of drug resistance.

### 3.8. Growth Factors

Multiple myeloma (MM) is known to rely strongly on the tumor cells on their microenvironment, which produces growth factors supporting survival and proliferation of MM cells. A detailed study provided an extensive description of myeloma growth factor (MGF) gene expression in the various cell populations of the BM of MM patients, which revealed an enhanced expression of MGF and MGFR genes during plasma cell differentiation [81]. Additionally, interleukin-6 has long been reported as a potent myeloma-cell growth factor in patients with aggressive MM [82]. Several reports affirming the clinical impact of hepatocyte growth factor, vascular endothelial growth factor, and antiapoptotic signaling pathways validate that growth factors are indeed crucial in maintaining the tumor progression in MM [83,84,85].

## 4. Components of the TME in the Light of Chromatin Remodeling

**Figure 2:**
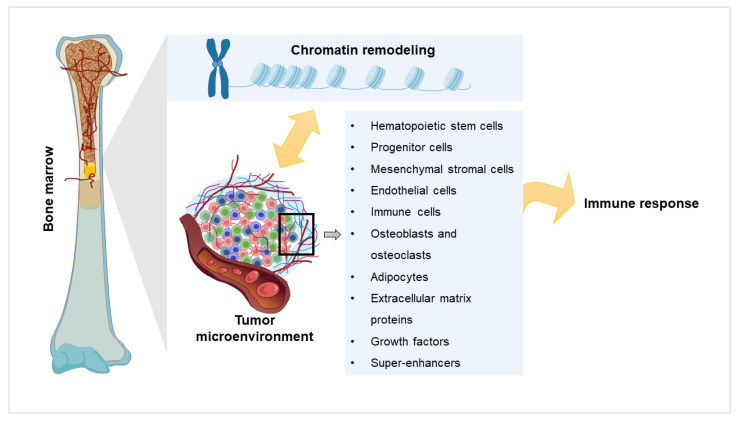
Crosstalk of the components of the tumor microenvironment with chromatin remodeling network.

Epigenetic modifications are well known to contribute to cancer development and progression [86]. Epigenetic modifications or changes lead to epigenetic marks, which specifically characterize different cells, such as tumor-associated macrophages, lymphocytes, monocytes, etc., of the tumor microenvironment [87]. While there are many reports in the cancer research field about epigenetic modifications, rising evidence signifies their role in developing a favorable TME [6]. The crosstalk of the components of the tumor microenvironment with chromatin remodeling network (summarized in Figure 2) has been reviewed in the next few paragraphs.

Hematopoietic stem cells are known to be regulated at the level of chromatin remodeling in various areas of research. Reports indicate that glucocorticoid hormone induces chromatin remodeling and, in turn, enhances recruitment of human hematopoietic stem cells and their engraftment [88]. The BAF45a/PHF10 subunit of SWI/SNF-like chromatin remodeling complexes has been reported to be crucial for maintenance of hematopoietic stem cell [89]. The chromatin remodeler BPTF is reported to promote maintenance of adult hematopoietic stem cells via activation of a stemness gene-expression pathway [90]. Chromatin remodeler Znhit1 is known to maintain hematopoietic stem cell quiescence by determining the access to distal enhancers [91]. Stem cell self-renewal is also known to be regulated by chromatin remodeling factors [92]. Chromatin remodeling factor Mll1 has been reported to be needed for neurogenesis from postnatal neural stem cells [93]. Embryonic stem cell self-renewal and pluripotency is regulated by embryonic stem cell chromatin remodeling complex, esBAF [94]. The maintenance of undifferentiated mouse embryonic stem cells is possible due to BAF250B-associated SWI/SNF chromatin remodeling complex [95]. Human embryonic stem cells undergoing pancreatic differentiation have been reported to be coordinated by the dynamic chromatin remodeling mediated by polycomb proteins [96]. In epidermal progenitor cells, p63 and Brg1 regulate the higher-order chromatin remodeling during their differentiation [97].

Mesenchymal cells are an integral part of MM TME and they are under epigenetic control by various means. It has been reported that, in human mesenchymal stem cells, exposure to unfavorable microenvironments leads to their extensive chromatin remodeling [98]. Recent reports show that chromatin remodeling agent trichostatin A in the liver cells modulates the differentiation process of human mesenchymal stem cells (hMSCs) in bone marrow [99]. PBAF-dependent chromatin remodeling and BMP/TGF-β signaling are important events that happen during mesenchymal stromal cell osteolineage differentiation due to the effect of Pbrm1 [100]. The cell growth arrest, apoptosis, and senescence of rat mesenchymal stem cells involves Brg1, an important chromatin remodeling factor [101]. Epigenetic control of mesenchymal stem cells also, by and large, regulates cell senescence. Several studies have reported that the chromatin remodeling complex factor BRG1 ATPase, which reportedly alters nucleosome structure by hindering histone protein and DNA interaction, promotes senescence of mesenchymal stem cells through influencing the RB–P53 molecular pathway [102] and, thus, might be crucial in silencing of NANOG protein, thus altering the expression levels of several chromatin proteins affecting cellular physiology [103].

The endothelial cells are functionally relevant in many contexts, including angiogenesis, and there are ample reports of these cells being epigenetically regulated. For example, the NuRD chromatin remodeling complex enzyme CHD4 is known to inhibit transcription of hypoxia-induced endothelial Ripk3, thus preventing vascular rupture [104]. Endothelial differentiation is affected by chromatin remodeling factor Nox4 [105]. Angiogenic factor receptor VEGFR is regulated in breast cancer cells by its interaction with the SWI/SNF chromatin remodeling complex [106].

Many biochemical and molecular studies have uncovered important roles of epigenetic aberration in changing the nature of TAMs and, thus, epigenetic alteration of these TAMs has the molecular thrust to reprogram the tumor microenvironment in different tumors, as well as in myeloma (TME). These molecular events have a deep impact in transformation to an immunosuppressive environment from an antitumor environment. It is well known that cellular origins of macrophages are of two types, either they undergo differentiation from circulating monocytes in the blood stream or originate from the resident macrophages involved in early development of organs [107]. Activated macrophages can be subdivided into M1 (classically activated) and M2 (alternative activated) phenotypes [108]. Activation of classical M1 macrophage generally occurs in response to bacterial infections and immune stimuli (lipopolysaccharide and interferon γ). M1 macrophages can also facilitate the innate immunity against tumor and parasites, resulting in inflammation by secretion of molecules such as tumor necrosis factor α, reactive nitrogen, and oxygen species. In addition, M1 macrophages evoke T-helper-1 (Th1) responses [109]. In contrast, M2 macrophages are responsive to cytokines such as IL-4, IL-13, IL-10, and glucocorticoid hormones; they play crucial roles in inflammatory response and wound healing and are majorly secreting immunosuppressive cytokines, such as IL-10 that promotes a Th2 immune response [110,111]. Although TAMs were believed to have an M2-like phenotype, promoting cancer growth and metastasis [112], recent evidence suggests that the TAMs may undergo M1–M2 transition [113]. Tumors contain M1 macrophages initially [114,115], which, on progression, switch to an M2-like characteristic [116,117]. It has been shown specifically that post-translational modifications of histone proteins associated with the inflammation-related genes influences the epigenetic machinery that controls activation of M1 and/or M2. The development and formation of macrophage phenotype is governed by the alterations in histone acetylation by acetyltransferases, and deacetylases and histone methylation by methyltransferases and demethylases. Furthermore, genetic regulations of macrophage gene expression occur mainly in the enhancers of related genes and during differentiation of macrophage. Moreover, macrophage promoters and lineage-specific enhancers undergo histone modifications [118]. Active enhancers are characterized by accumulation of H3K27ac [119]. It has also been reported that M2 activation is majorly associated with strong association of histone methyl transferases, resulting in the repression of M1 phenotype and promoting the transcription of M2 genes [120].

The role of chromatin modulators in osteoclast and osteoblast dynamics in TME is very interesting. Dpy30 has a role in osteoclast differentiation and function [121]. MITF and PU. 1 recruit p38 MAPK and NFATc1 to target downstream genes during osteoclast differentiation [122]. Histone deacetylases regulate osteoclast differentiation and skeletal maintenance [123]. Transcriptional activation for osteogenesis and odontogenesis is affected by Baf45a-mediated chromatin remodeling [124]. Moreover, HIF-1α has been known to disrupt the osteoblasts and osteoclasts balance in bone remodeling by upregulation of OPG expression [125].

Extensive chromatin remodeling during early adipogenesis indicates that TME adipocytes could essentially be epigenetically regulating the tumor [126]. The SWI/SNF protein BAF57 is reported to control adipogenesis [127]. Ucp1 expression in murine adipose tissue has been known to be controlled by Ucp1 enhancer methylation and chromatin remodeling [128]. EBF2 regulates brown adipogenesis transcriptionally with the help of histone reader DPF3 and chromatin remodeling complex BAF [129]. 

Evidence that epigenetics play a role in modulating extracellular matrix protein expression includes reports that co-operation of SWI/SNF and transcription factors is highly required to control the extracellular-matrix-regulated gene expression [130]. Moreover, the NuRD chromatin-remodeling enzyme CHD4 elevates embryonic vascular integrity by regulating extracellular matrix proteolysis [131]. Extracellular matrix remodeling is also promoted by the inhibition of histone deacetylase activity in human endometrial stromal cells [132].

## 5. Super-Enhancers Affect Chromatin Remodeling and Bone Marrow Microenvironment in Myeloma

Multiple myeloma progression is affected by activity of several super-enhancers, which are one of the most important cis-regulatory DNA elements containing several binding motifs for transcription factors such as Myc, IRF4, etc. [133]. These transcription factors, by binding to super-enhancers, modulate the chromatin accessibility of myeloma cells. A recent study showed that aberrant expression of cyclinD2 might provide a way for the identification and characterization of novel super-enhancer-associated oncogenes, which are biologically relevant in myeloma [134]. Some of these super-enhancer-based dependencies may be exploited for potential therapeutic targets. Chromatin regulatory factors, such as transcriptional coactivator BRD4, are inhibited by the BET domain inhibitor, namely JQ1, which resulted in substantial loss of BRD4 in MYC super-enhancers and associated transcriptional anomalies [135]. A comprehensive combinatorial study revealed aberrant transcription factor regulation network and the epigenetic changes in MM by analysis of myeloma gene expression, openness of chromatin, and enhancer landscape [136]. Many important gene loci, earlier linked to myeloma progression, depict increased super-enhancer activity, as well as gene expression, for example, genes involved in bone marrow microenvironment, such as IL6S, CD200, KIT, ITGA4 CXCR4, etc. [136]. Most importantly, super-enhancers, as well as genes, identified in several steps of molecular pathways, such as NF-kB pathway, p53 signaling, mTOR signaling, cancer stem cell pathway, and NOTCH pathway, in myeloma maintain the crosstalk within the myeloma microenvironment, resulting in the activation of chromatin regulatory network in myeloma [14].

## 6. Conclusions

As per the current literature available, it is very clear that chromatin remodeling in the light of the tumor microenvironment has deep impact in multiple myeloma, as well as other hematological malignancies. Thus, a careful understanding of the regulatory circuitry, which includes several DNA modifying elements governing the crosstalk between the tumor microenvironment and chromatin remodeling factors and complexes affecting the molecular landscape of multiple myeloma, will help us to identify novel oncogenic mechanisms underlying myeloma initiation and progression, and might provide novel therapeutic opportunities in the future.
